# Supplementary material for: Enhanced Carbonylation of Photosynthetic and Glycolytic Proteins in Antibiotic Timentin-Treated Tobacco In Vitro Shoot Culture
Source: Plants (Basel). 2022 Jun 14;11(12):1572. doi: 10.3390/plants11121572 (PMC9228549; doi:10.3390/plants11121572)
Supplement: Supplementary file 1 [file plants-11-01572-s001.zip › plants-1752580-Supplementary.pdf]

plants

MDPI

Supplementary Materials

Enhanced Carbonylation of Photosynthetic and Glycolytic Proteins in Antibiotic Timentin-Treated Tobacco In Vitro Shoot Culture

Elena Andriūnaitė, Rytis Rugienius, Inga Tamošiūnė, Perttu Haimi, Jurgita Vinskienė and Danas Baniulis *

Institute of Horticulture, Lithuanian Research Centre for Agriculture and Forestry, Kaunas Str. 30, Kaunas reg., Babtai 54333, Lithuania; elena.andriunaite@lammc.lt (E.A.); rytis.rugienius@lammc.lt (R.R.); inga.tamosiune@lammc.lt (I.T.); perttu-juhani.haimi@lammc.lt (P.H.); jurgita.vinskiene@lammc.lt (J.V.)
* Correspondence: danas.baniulis@lammc.lt

Table S1. Variation of superoxide ($O_2^{\bullet-}$), hydrogen peroxide (H_2O_2), and protein carbonyl modification content in tobacco in vitro shoot tissues during the propagation cycle.

**Time, days**	**$O_2^{\bullet-}$, µmol g^{-1} F.W.**		**H_2O_2, µmol g^{-1} F.W.**		**Carbonyl, nmol mg^{-1} protein**	
	**TC**	**PA**	**TC**	**PA**	**TC**	**PA**
1	0.83 ± 0.02^{a}	0.95 ± 0.05^{a}	19.6 ± 0.5^{ac}	21.3 ± 0.4^{a}	4.0 ± 0.1^{a}	6.6 ± 0.9^{b}
	n=5	n=6	n=5	n=5	n=4	n=4
4	1.10 ± 0.05^{ac}	0.89 ± 0.02^{a}	17.5 ± 0.5^{ab}	19.3 ± 1.0^{ac}	10.1 ± 1.0^{c}	10.0 ± 0.6^{bc}
	n=5	n=7	n=5	n=8	n=4	n=4
7	0.98 ± 0.02^{a}	1.38 ± 0.09^{bc}	17.0 ± 0.7^{b}	15.6 ± 0.6^{b}	8.5 ± 0.5^{c}	13.0 ± 1.1^{d}
	n=10	n=15	n=12	n=15	n=4	n=4
14	1.56 ± 0.02^{bd}	1.89 ± 0.03^{e}	14.8 ± 1.1^{b}	17.1 ± 0.7^{bc}	6.2 ± 0.3^{a}	10.7 ± 1.3^{cd}
	n=9	n=7	n=5	n=11	n=3	n=3
21	1.41 ± 0.04^{bc}	1.79 ± 0.07^{de}	14.8 ± 0.4^{b}	15.4 ± 0.5^{bc}	9.4 ± 1.5^{c}	10.8 ± 0.9^{cd}
	n=5	n=5	n=5	n=5	n=3	n=3

$O_2^{\bullet-}$ and H_2O_2 content was assessed using NBT and DAB staining, respectively, as described in the Materials and Methods section. Data presented as the mean ± standard error of the mean of n replicates including 3-5 shoot pooled sample each. Different letters denote significant differences ($p < 0.05$). Time corresponds to days after shoot transfer to fresh medium. Abbreviations: F.W. – fresh weight, TC – treatment control, PA – timentin-treated.

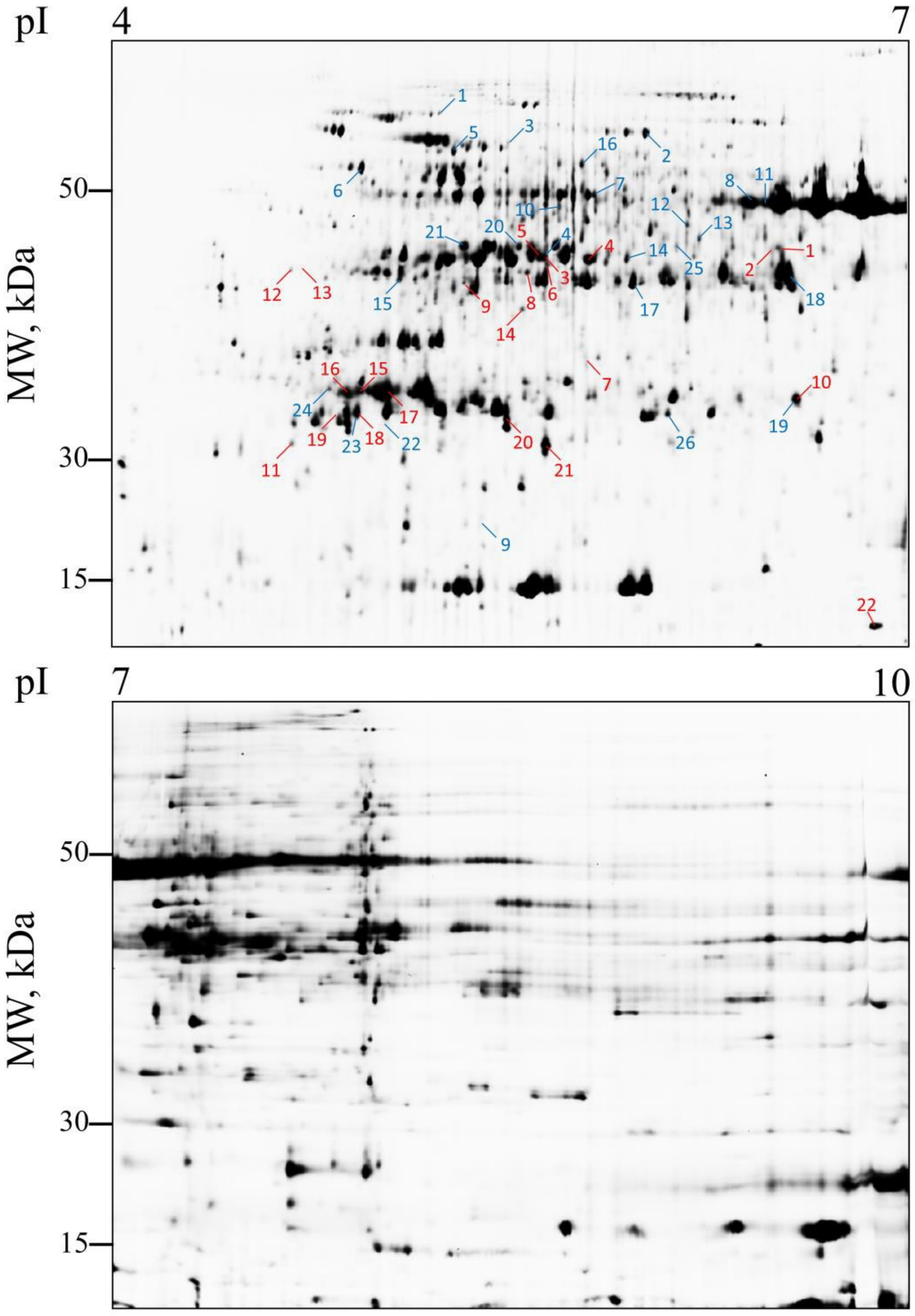

Figure S1. Results of tobacco shoot in vitro culture proteome analysis using differential gel electrophoresis. The representative gel image illustrates separation of pooled sample proteins labeled with Cy2 dye. Isoelectrical focusing was performed at two linear ranges of pH 4–7 and pH7–10 for protein expression analysis, and only acidic range of pH7-10 was used for protein carbonylation analysis. Numbers indicate identified proteoforms differentially expressed between the control tobacco shoot samples collected at the early and late phase (after one and three weeks of cultivation, respectively) of the propagation cycle (red) or proteins differentially carbonylated between control and timentin-treated shoot samples (blue) as indicated in Tables 1–2 and illustrated in Appendix A Figures A2–A3. Abbreviations: MW – molecular weight; pI—isoelectricpoint

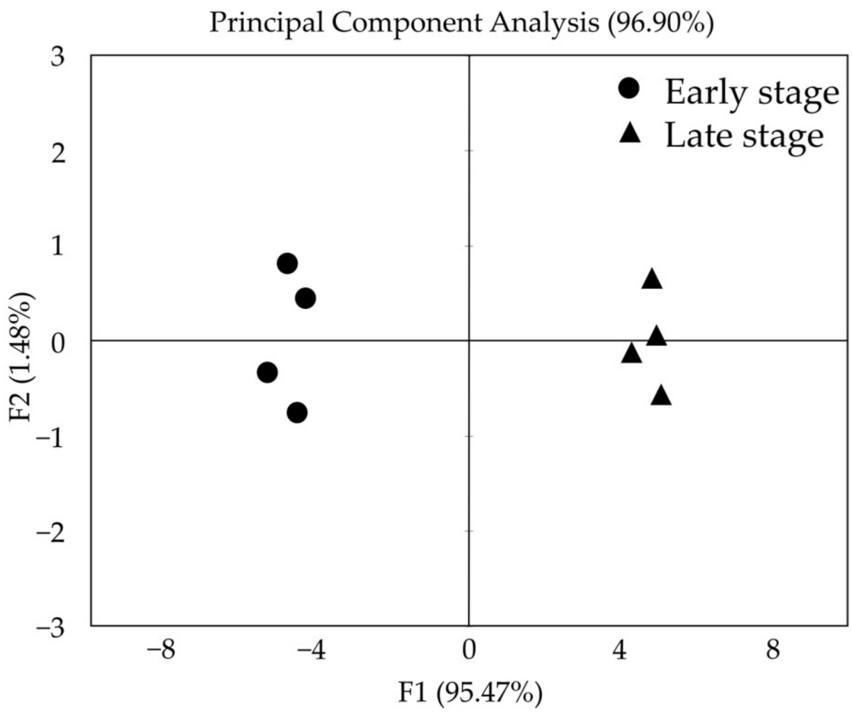

Figure S2. Principal component analysis of protein abundance data of timentin-treated tobacco in vitro shoot samples collected at the early and late stage (after one and three weeks of cultivation, respectively) of the propagation cycle and analyzed using differential gel electrophoresis.

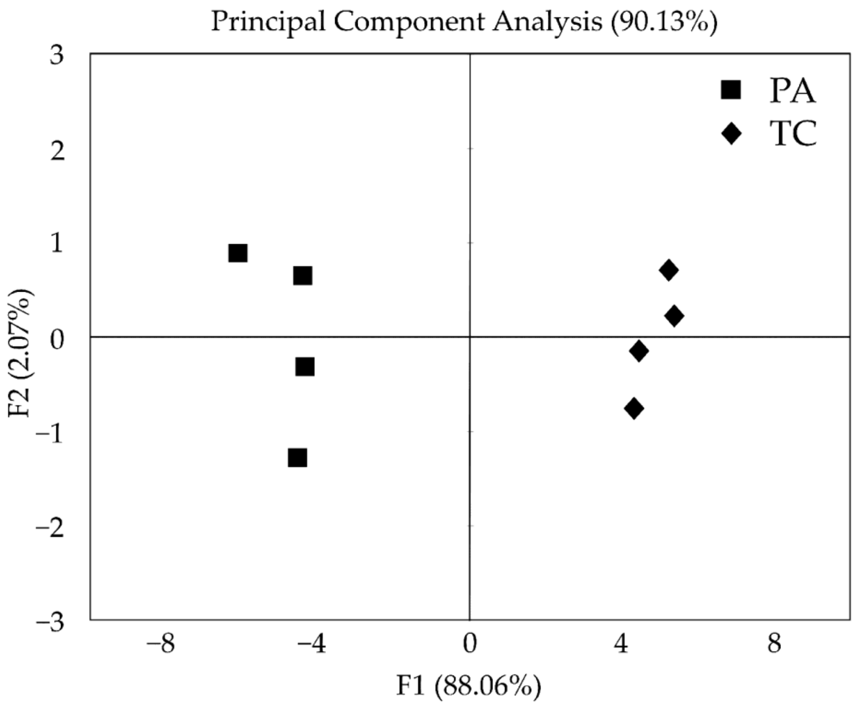

Figure S3. Principal component analysis of protein carbonylation data of control (TC) and timentin-treated (PA) tobacco in vitro shoot samples, collected after one week of cultivation (day 7) and analyzed using differential gel electrophoresis.

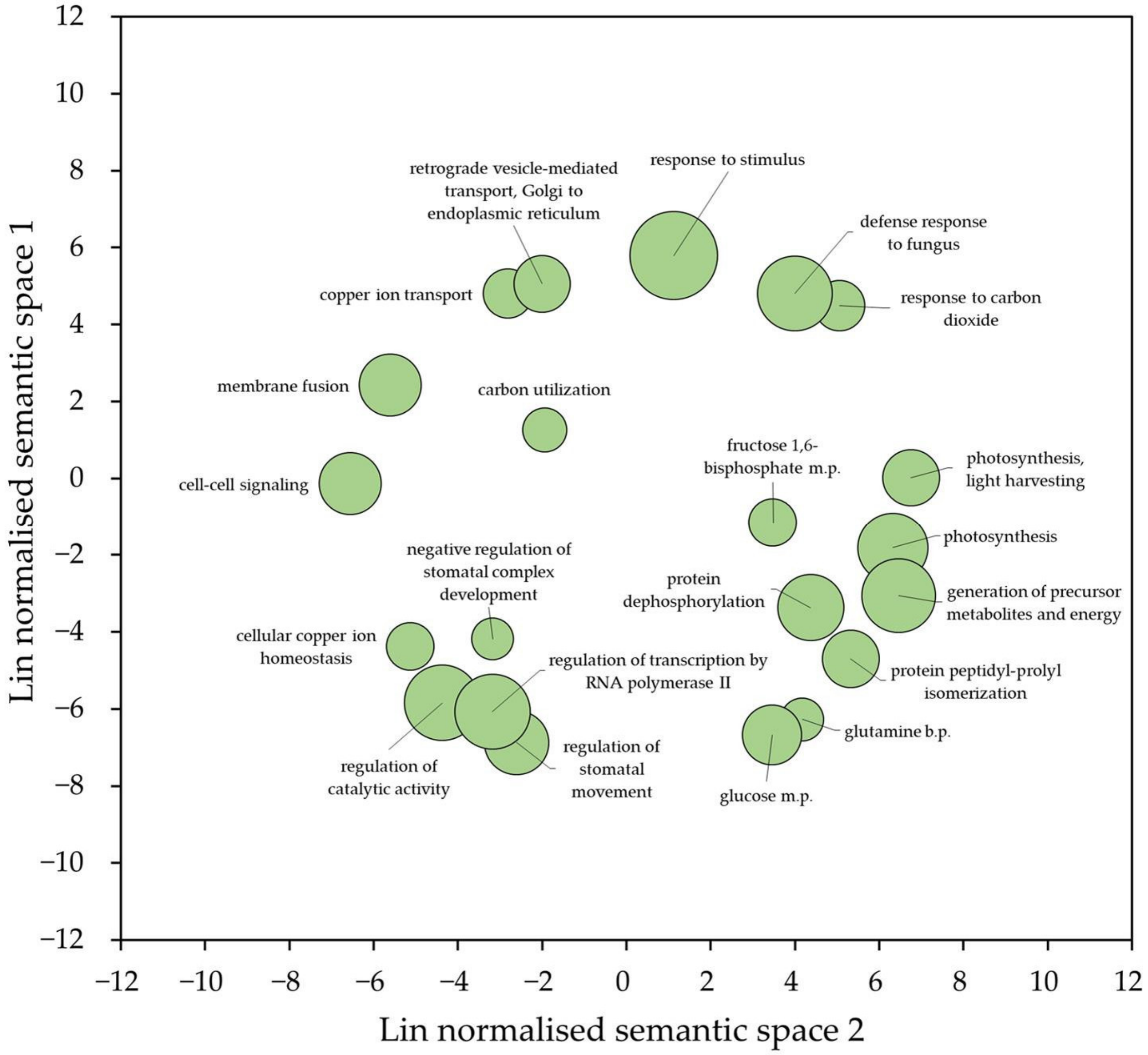

Figure S4. Summary of GO terms associated with proteoforms differentially expressed between timentin-treated tobacco in vitro shoot samples collected at the early and late stages of the propagation cycle. GO terms are represented by circles and are plotted according to semantic similarities to other GO terms using the Revigo server. Sizes of the plotted circles are scaled by the number of GO terms they represent. Abbreviations: m.p.—metabolic process, base—biosynthetic process.

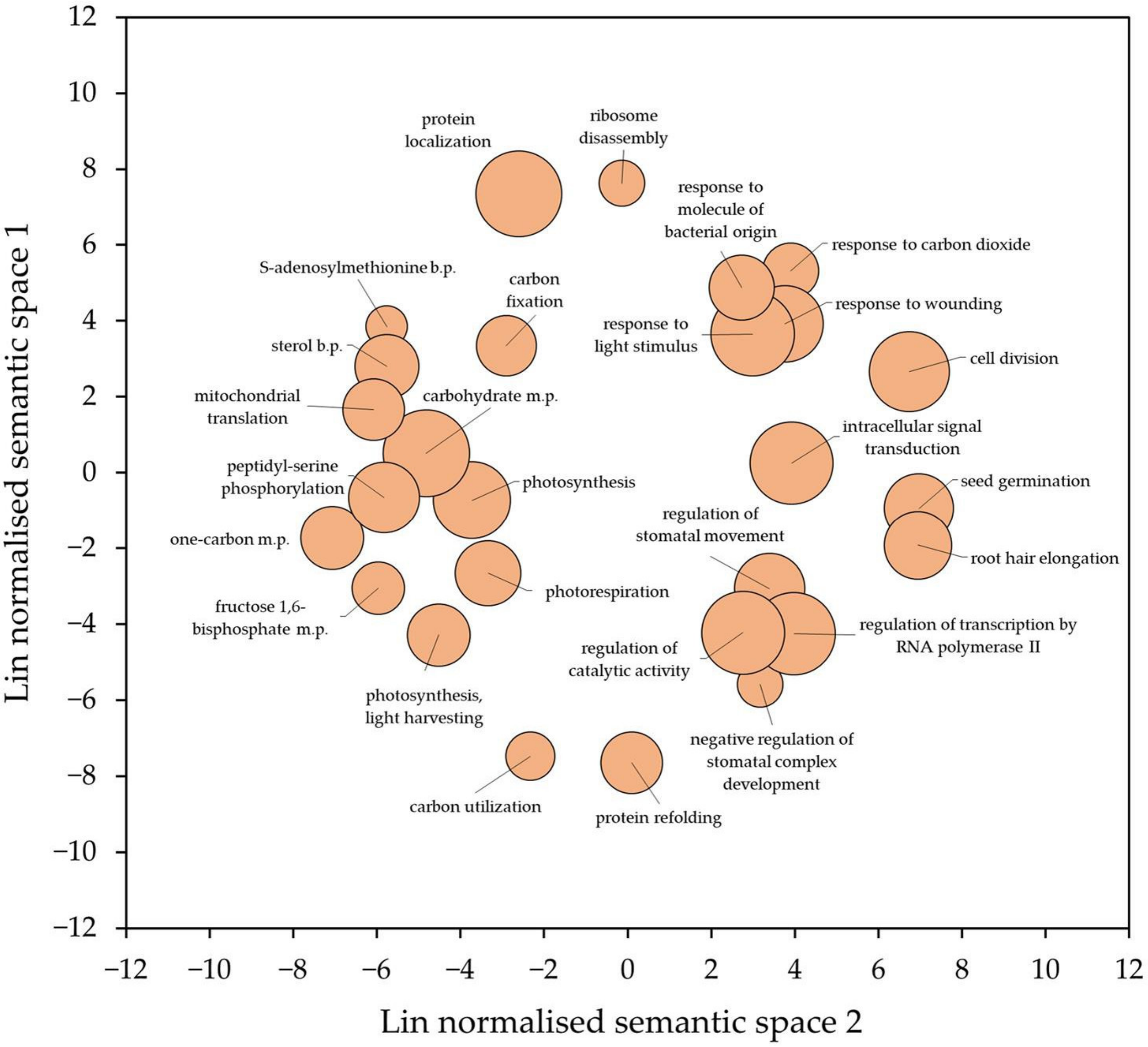

Figure S5. Summary of GO terms associated with proteoforms differentially carbonylated between timentin-treated (PA) and control (TC) tobacco in vitro shoot samples. GO terms are represented by circles and are plotted according to semantic similarities to other GO terms using the Revigo server. Sizes of the plotted circles are scaled by the number of GO terms they represent. Abbreviations: m.p.—metabolic process, b.s.—biosynthetic process.
